# Supplementary material for: Proposal for a Global Adherence Scale for Acute Conditions (GASAC): A prospective cohort study in two emergency departments
Source: PLoS One. 2019 Dec 10;14(12):e0215415. doi: 10.1371/journal.pone.0215415 (PMC6903735; doi:10.1371/journal.pone.0215415)
Supplement: S1 File — (PDF) [file pone.0215415.s001.pdf]

Appendix 1

Examples of calculation of the GASAC score:

- Case n°1 with answers to all 6 questions:

WI=3

WII=3

WIII=4

X=4

Y=3

Z=3

Total score = [(3+3+4+4+3+3) - 6] / (3*6) = 0.78

- Case n°2 with replies to the drug sub-section only (the physician gave no other prescriptions or advice):

WI=1

WII=4

WIII=3

X= not applicable (not scored)

Y= not applicable (not scored)

Z= not applicable (not scored)

Total score = [(1+4+3) - 3] / (3*3) = 0.56
