# Supplementary material for: Proposal for a Global Adherence Scale for Acute Conditions (GASAC): A prospective cohort study in two emergency departments
Source: PLoS One. 2019 Dec 10;14(12):e0215415. doi: 10.1371/journal.pone.0215415 (PMC6903735; doi:10.1371/journal.pone.0215415)
Supplement: S2 File — (PDF) [file pone.0215415.s002.pdf]

| **Questionnaire d'Adhérence Globale aux prescriptions médicales (GASAC)**<br>*Réponses possibles pour chaque question: **Non, pas du tout =1 ; En partie=2 ; Plutôt oui =3 ; Oui, tout à fait= 4*** | |
|---|---|
| **A- Adhérence médicamenteuse**<br><br>I- Avez-vous pris l'ensemble du traitement prescrit ?<br><br>II- Avez-vous respecté les doses prescrites ?<br><br>III- Avez-vous respecté les modalités de prises ? (heure, à jeun, selon les repas) | Sous-Total<br><br>x |
| **B- Adhérence non médicamenteuse**<br><br>IV- Si votre médecin vous a prescrit des examens complémentaires (imagerie, biologie) ou une consultation spécialisée, les avez-vous realisés ? Si "non", non comptabilisé. | Sous-Total<br><br>y |
| **C- Adhérence aux conseils hygiéno-diététiques**<br><br>V- Si votre médecin vous donné des conseils ? (ex: alimentation, activités habituelles; activité physique, activité professionnelle, tabac, alcool, hydratation, entourage), les avez-vous appliqués ou avez-vous modifié certaines habitudes suite à la consultation ?<br><br>Si "non", non comptabilisé | Sous-total<br><br>z |
| **D- Adhérence à l'accès au système de soins**<br><br>VI- Si votre médecin vous a donné des consignes de surveillance et des conseils sur "quand reconsulter", les avez-vous appliqués ? Si "non", non comptabilisé | Sous-total<br><br>w |
| Score total = [(x+y+z+w) – nombre de questions comptabilisées]/(3 * nombre de questions comptabilisées) | **Score total**<br>(entre 0 et 1). |

***Questions subsidiaires non comptabilisées***

**Vous n'avez pas pris tout votre traitement**

*a- Parce que vous l'avez oublié: ................................................................................ oui / non*
*b- Parce que le traitement est trop complexe: ............................................................ oui / non*
*c- Parce qu'il provoque des effets secondaires: ......................................................... oui / non*
*d- Parce vos médicaments vous font plus de mal que de bien : ................................. oui / non*
*e- Parce vous pensiez qu'il n'était pas utile ou adapté: ............................................ oui / non*
*f- Parce que vous sentiez déjà une amélioration : ......................................................oui / non*
*g- Parce que vous n'aviez pas mal: ............................................................................oui / non*
*h- Parce que vous n'êtes pas allé chercher les médicaments à la pharmacie :………......oui / non*
*Si oui : pourquoi ?......................................................................................................*
*i-* Avez-vous pris d'autres médicaments que ceux prescrits pas votre médecin ?....... *oui / non*
*Si oui, lesquels ?.........................................................................................................*
*j- Ces médicaments ont-ils été prescrits par un autre médecin ?...............................oui / non*
*k- Votre médecin vous a-t-il prescrit des examens biologiques ? …...........................oui / non*
*l- Votre médecin vous a-t-il prescrit une imagerie ?.................................................. oui / non*
*m- Votre médecin vous a-t-il prescrit un RDV chez un spécialiste ?......................... oui / non*
*n- Votre médecin vous a-t-il donné un RDV de suivi avec lui ?................................. oui / non*
*o- Certaines informations ou conseils pouvez intéresser vos proches ? ….……………oui / non*
*p- Si oui, les avez-vous transmis ? ...................... ..................................................... oui / non*
*q- Avez-vous eu besoin d'un avis médical pour le même problème ? ........................ oui / non*
*r- Si oui, avez-vous consulté à nouveau votre médecin traitant ? ..............................oui / non*
*s- Un autre médecin ? ...... ............................................................ ..............................oui / non*
*t- Un service d'urgences ? ........................... ...............................................................oui / non*
*u- Avez-vous appelé le centre 15 ? ................ ........................... ..............................oui / non*
*v- Votre attitude était-elle conforme aux conseils donnés par votre médecin ?...........oui / non*
