# Supplementary material for: Proposal for a Global Adherence Scale for Acute Conditions (GASAC): A prospective cohort study in two emergency departments
Source: PLoS One. 2019 Dec 10;14(12):e0215415. doi: 10.1371/journal.pone.0215415 (PMC6903735; doi:10.1371/journal.pone.0215415)
Supplement: S4 File — (PDF) [file pone.0215415.s004.pdf]

PROTOCOLE

INFORMATIONS GENERALES

**Titre :** Etat des lieux des niveaux de communication médecin malade (CMM) lors d'une consultation pour pathologie aigue dans un service d'Urgences Médicales en vue d'améliorer les pratiques. Impact sur l'observance globale et la satisfaction des patients consultant pour entorse de cheville ou pyélonéphrite aigue dans un service d'urgences.
**Méthodologie :** Évaluation de pratiques sans procédure particulière de surveillance par une enquête téléphonique : étude observationnelle monocentrique prospective à partir de données déclaratives
**Investigateurs coordonnateurs autorisés à signer le protocole:** Dr Mélanie SUSTERSIC, Pr Jean-Luc BOSSON
**Investigateurs coordonnateurs :** Amélie DUVERT, Laure GONNET
**Investigateurs :** Médecins Urgentistes (Service d'Urgences de la Clinique Mutualiste à Grenoble)
**Intervenants :** Pr Jean-Luc BOSSON (CIC, TIMC IMAG), Dr Aurélie Gauchet (Laboratoire Inter universitaire de Psychologie, UPMF), Pr Benoît ALLENET (TIMC IMAG).
**Centre coordonnateur de l'étude : CIC**<br>Centre d'Investigation Clinique – Inserm003<br>CHU de Grenoble, 38043 Grenoble Cedex 09<br>Tel : 04 76 76 92 60 Fax : 04 76 76 92 62

PROTOCOLE – RESUME
**Investigateurs coordonnateurs autorisés à signer le protocole :** Dr Mélanie SUSTERSIC, Pr Jean-Luc BOSSON
**Investigateurs coordonnateurs:** Amélie DUVERT, Laure GONNET
**Investigateurs :** Médecins Urgentistes (Service d'Urgences de la Clinique Mutualiste à Grenoble)
**Intervenants :** Pr. Jean-Luc BOSSON, Pr Benoît ALLENET, Dr Aurélie GAUCHET
**Méthodologie :** Étude observationnelle monocentrique prospective
**Objectif principal :** Faire un état des lieux des niveaux de communication médecin malade (CMM), d'observance globale (OG) et de satisfaction des patients dans un service d'urgence en vue d'améliorer les pratiques.<br><br>**Critère de jugement principal :** Mesure des scores de CMM, d'OG et de la satisfaction des patients entre J7 et J10 au décours d'une consultation dans un service d'urgences (pour 2 motifs fréquents : entorse de cheville et pyélonéphrite aiguë).

Objectifs secondaires :
1. Évaluer les propriétés psychométriques des scores de CMM et d'OG créés pour l'étude ;
2. Évaluer la corrélation entre l'observance thérapeutique de Girerd et la sous dimension « observance médicamenteuse » du score d'OG créé pour l'étude ;
3. Évaluer la corrélation entre l'observance de Girerd et l'OG des patients ;
4. Évaluer la corrélation entre la CMM et l'OG;
5. Évaluer la corrélation entre la CMM et la satisfaction ;
6. Évaluer la corrélation entre l'OG et la satisfaction.

Critères de jugement secondaires :
1. Mesure des propriétés psychométriques (coefficient de Cronbach) des scores de CMM et d'OG. Les 2 scores seront mesurés lors d'une enquête téléphonique entre J7 et J10 après la consultation.
2. Mesure de la corrélation entre le score d'observance thérapeutique de Girerd et la sous-dimension « observance médicamenteuse » du score d'OG créé pour l'étude ;
3. Mesure de la corrélation entre le score d'observance de Girerd et le score d'OG ;
4. Mesure de la corrélation entre le score de CMM et le score d'OG ;
5. Mesure de la corrélation entre le score de CMM et le score satisfaction ;
6. Mesure de la corrélation entre le score d'OG et le score de satisfaction.

Critères d'inclusion principaux : majeur, lettré, ayant consulté dans un service d'urgence pour une des pathologies choisies (entorse de cheville ou pyélonéphrite aigue) et pouvant être joint par téléphone dans les dix jours qui suivent.

Nombre total de sujets à inclure : 200 pour obtenir une précision suffisante dans l'étude descriptive des objectifs principaux (IC95% de l'ordre de ± 5 %)

Durée totale de l'étude : 4 mois

Durée de l'étude par sujet : 7 à 10 jours

I. JUSTIFICATION DE L'ETUDE, INTERET ET CONTEXTE GENERAL

La loi HPST (Loi n°2009-879 du 21 juillet 2009 portant sur la réforme de l'hôpital et relative aux patients, à la santé et aux territoires) (1) instaure l'obligation de développement professionnel continu (DPC) des professionnels de santé. Selon l'article 59 de cette loi, le DPC a pour objectifs « l'évaluation des pratiques professionnelles, le perfectionnement des connaissances, l'amélioration de la qualité et de la sécurité des soins ainsi que la prise en compte des priorités de santé publique et de la maîtrise médicalisée des dépenses de santé ».

Plus particulièrement, l'arrêté du 26 février 2013 fixe deux orientations nationales particulières pour l'année 2013 :

- « contribuer à l'amélioration de la prise en charge des patients » (via notamment « la connaissance de l'état de santé de la patientèle » et « les programmes d'études cliniques et épidémiologiques visant à évaluer des pratiques »)
- « contribuer à l'amélioration de la relation entre professionnels de santé et patients » (via notamment « le développement de l'information et de l'autonomie du patient ; » et avec des DPC ayant pour objectif « de favoriser le bon usage et l'observance des traitements » ; ainsi que « la formation à la relation professionnel de santé-patient ou au partenariat soignant-soigné. »)

Il n'est pas étonnant que ces deux concepts d'observance thérapeutique (OT) et de communication médecin-malade (CMM) soient à l'ordre du jour des objectifs nationaux de Santé Publique.

En effet, concernant l'OT, selon Haynes « [elle] se définit par l'importance avec laquelle les comportements (en termes de prise de médicaments, de suivi de régime ou de changements de mode de vie) d'un individu coïncident avec les conseils médicaux ou de santé » (3). L'évaluation de l'observance a fait le sujet de nombreuses publications récentes. Améliorer l'observance est une des actions permettant d'atteindre l'un des objectifs principaux des soignants : l'amélioration de l'état de santé et de la qualité de vie des patients (4). Pour l'OMS « Optimiser l'observance médicamenteuse aurait plus d'impact en terme de santé mondiale que le développement de nouveaux médicaments »(5).

L’importance de l’OT a été soulignée, par l’OMS notamment, pour les maladies chroniques principalement (6). Qu’il s’agisse de pathologie aigue ou chronique, il est pourtant essentiel que tous les patients puissent comprendre et connaître la pathologie et le traitement qui les amènent à consulter (7,8,9).

De plus, la « non observance » a de nombreuses conséquences au niveau médical et économique (4,10).

L’observance est donc une dimension importante qui reflète le comportement du patient en termes de suivi des prescriptions médicamenteuses et des conseils hygiéno-diététiques et ainsi qui est un des reflets de la qualité d’une consultation.

Cependant l’observance est un « phénomène instable, dynamique et modifiable » (11). De nombreux facteurs l’influençant ont été identifiés. L’OMS en répertorie 5 classes, dont ceux liés au système de soins qui comprennent la qualité de la relation thérapeutique (5). De même pour de nombreux psychologues en santé (12), c’est la communication médecin-malade au cours de la consultation qui est cruciale dans la formation de l’observance ou de l’inobservance (11). En effet différents paramètres entrent en compte : la délivrance de l’information et la compréhension de celle-ci par le patient ainsi que la dimension affective de la relation. « Le sentiment d’être compris et informé améliore la compliance » (13).
Les relations thérapeutiques « pauvres » seraient un facteur de non adhésion (4, 14,15,16).

Pouvoir évaluer ces deux paramètres (OT et CMM) facilement, nous paraît donc crucial dans une démarche de DPC, en visant à terme leur amélioration.

Cependant, il n’existe pas dans la littérature de score générique évaluant la communication médecin-malade ni l’observance thérapeutique, pouvant être comparables d’une pathologie à l’autre. Deux scores génériques, un de CMM et un d’observance globale (OG) ont donc été créés à cette fin, par la même équipe de chercheur, lors d’une phase préparatoire (détails dans le protocole).

Notre étude se déroulera en 2 phases dont seule la première phase fait l’objet du présent protocole :

- Une première phase (protocole ci-après) ayant pour objectif d’une part de faire un état des lieux des niveaux de communication médecin malade (CMM),

d'observance globale (OG) et de satisfaction des patients dans un service d'urgences en vue d'améliorer les pratiques ; et d'autre part, l'évaluation des qualités intrinsèques de ces 2 scores (Coefficient de Cronbach). Ces deux scores ont pour but d'être utilisés comme indicateur de la qualité des pratiques professionnelles pour toute situation clinique.

Nous évaluerons dans le même temps les liens de corrélation entre l'OG, la CMM et la satisfaction des patients à l'état basal, dans un service d'urgences. En effet, si ce lien existe, tout programme visant à l'amélioration de la CMM, permettrait donc de ce fait d'améliorer l'OG.

- Puis un deuxième projet (lancement prévu pour l'hiver 2014), aura pour objectif d'évaluer l'amélioration des pratiques professionnelles par l'usage de deux Fiches d'Information pour les Patients (entorse de la cheville et pyélonéphrite aigue) dans un service d'urgences. Il s'agira d'une étude de type avant/après qui aura pour but de mesurer l'impact sur la CMM, l'OG et la satisfaction des patients de ces deux FIP délivrées en complément de l'information orale, au cours de la consultation. Notre projet actuel permettra de décrire les différents scores, de les améliorer en cas de redondance d'information et de justifier précisément les hypothèses du deuxième projet interventionnel de type avant-après.

En effet, l'utilisation de Fiches Information Patient (FIP) est d'usage courant et est l'une des pistes actuelles dans l'amélioration globale des soins ainsi qu'en éducation thérapeutique (17). Notre postulat est que la distribution d'une information écrite (les FIP), délivrée en complément de l'information orale, dans des situations très courantes (les consultations aux Urgences pour des pathologies aigues et nécessitant un prise en charge médicamenteuse et non-médicamenteuse), permettrait d'améliorer la CMM et l'OG. Si un lien de corrélation était établi entre les deux, il suffirait alors de mesurer isolément la CMM pour obtenir un reflet de la qualité de la prise en charge.

Les FIP que nous utiliserons sont issues d'un premier travail, débuté à l'université de médecine de Grenoble en 2007 (18, 19) qui avait permis d'élaborer une méthodologie d'aide à la réalisation de fiches d'information patient (FIP) puis 125 FIP concernant des pathologies ou des situations courantes en soins primaires.

Enfin, ce sont les spécificités d'un service d'Urgences qui nous ont conduits à choisir un tel service pour la réalisation de cet axe de recherche. En effet, la rapidité des consultations, la nécessité de prendre en charge « l'urgence » et non les « petits-bobos », le peu de temps de communication et d'échange possible entre le médecin et le patient, le temps d'attente, le manque de confidentialité dans la plupart des cas et surtout le problème de l'engorgement des services d'Urgences en France (20,21,22), sont autant de paramètres qui nous ont semblé rendre d'autant plus justifiés cette étude puis l'usage des FIP.

II. OBJECTIF DE L'ETUDE

II.1. Objectif principal :

Faire un état des lieux des niveaux de communication médecin malade (CMM), d'observance globale (OG) et de satisfaction des patients dans un service d'urgences en vue d'améliorer les pratiques.

Critère de jugement principal :

Mesure du score de CMM, d'OG et de la satisfaction des patients au décours d'une consultation dans un service d'urgences (pour entorse de cheville et pyélonéphrite aiguë). Les 2 scores seront mesurés lors d'une enquête téléphonique entre J7 et J10 après la consultation.

II.2. Objectifs secondaires :

1. Évaluer les propriétés psychométriques des scores de CMM et d'OG créés pour l'étude ;
2. Évaluer la corrélation entre l'observance thérapeutique de Girerd et la sous dimension « observance médicamenteuse » du score d'OG créé pour l'étude ;
3. Évaluer la corrélation entre l'observance de Girerd et l'OG des patients ;
4. Évaluer la corrélation entre la CMM et l'OG;
5. Évaluer la corrélation entre la CMM et la satisfaction ;
6. Évaluer la corrélation entre l'OG et la satisfaction.

Critères de jugement secondaires :

1. Mesure des propriétés psychométriques (coefficient de Cronbach) des scores de CMM et d'OG.
2. Mesure de la corrélation entre le score d'observance thérapeutique de Girerd et la sous-dimension « observance médicamenteuse » du score d'OG créé pour l'étude ;
3. Mesure de la corrélation entre le score d'observance de Girerd et le score d'OG ;
4. Mesure de la corrélation entre le score de CMM et le score d'OG ;
5. Mesure de la corrélation entre le score de CMM et le score satisfaction ;
6. Mesure de la corrélation entre le score d'OG et le score de satisfaction.

III. CONCEPTION DE LA RECHERCHE

III.1. Type d'étude

- Étude observationnelle
- Étude monocentrique
- Étude prospective à partir de données déclaratives
- Évaluation de pratiques sans procédure particulière de surveillance par une enquête téléphonique

III.2. Organisation générale de l'étude

III.2.1. Schéma expérimental

Enquête par questionnaire téléphonique avec mesure unique à J7-10 pour les 2 situations cliniques.

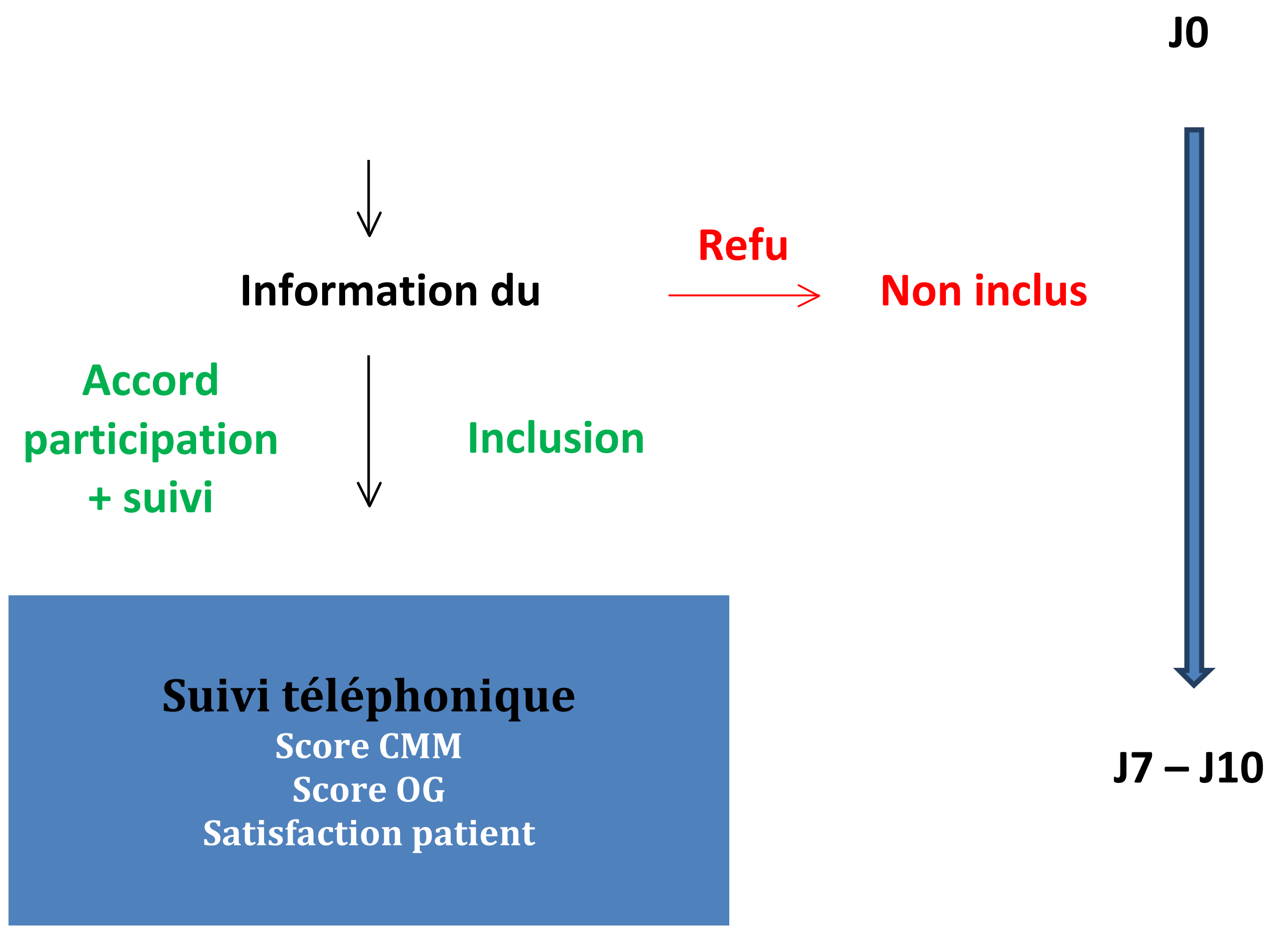

III.2.2. <u>Déroulement pratique de l'étude</u>

III.2.2.1. Phase préparatoire d'élaboration des questionnaires

Afin de pouvoir évaluer l'observance thérapeutique ainsi que la CMM, avec des scores pouvant être comparables d'une pathologie à l'autre, nous avons créé, lors d'une première phase, des scores d'observance globale et de CMM génériques, communs à toutes les pathologies (et non un score spécifique à chaque pathologie, comme existant dans la littérature).

III.2.2.1.1. <u>Le score d'observance (annexe 1)</u>

Il comprend 2 grandes parties :

- *La première partie A/ du questionnaire correspond au score d'observance thérapeutique de Girerd (23)*

Il s'agit d'un score validé, adapté à la langue Française et utilisé pour l'hypertension artérielle, très utilisé (il est la référence en matière de pathologies chroniques et est

en ligne sur le site de la Sécurité Sociale pour permettre aux médecins d'évaluer l'observance de leurs patients).
Ce score n'a pas été utilisé en situation aiguë sous sa version initiale. Malgré le contexte de la situation aiguë, le score reste inchangé avec les 6 mêmes questions et la même définition d'un patient non-observant (score ≥ 3).

- *La deuxième partie B/ correspond au score d'OG nouvellement créé.*

Étant donné qu'il n'existe pas dans la littérature de score générique d'observance concernant les pathologies aiguës, ni de score évaluant à la fois les aspect médicamenteux et non médicamenteux de l'observance pour les pathologies aiguës, nous avons donc élaboré, un nouveau score composite. Nous nous sommes appuyés sur divers questionnaires dont un score validé utilisé dans l'évaluation de l'observance au traitement du VIH par anti-rétro-viraux (13), le SATMED-Q (24) et des questionnaires de l'AHRQ (25).
Nous appellerons le score d'observance nouvellement créé, score d'observance global (OG) par opposition au score d'observance médicamenteuse de Girerd ou au concept d'OT défini précédemment. Ce score d'OG comprend 4 sous-dimensions :
- l'observance des prescriptions médicamenteuses (même cible que le score de Girerd) ;
- l'observance des prescriptions autres que médicamenteuses (examens complémentaires) ;
- l'observance des règles hygiéno-diététiques ;
- le comportement en termes de consommation de soins (reconsultations…).

Nous mesurons le score de Girerd en première partie, afin de nous appuyer sur un score validé. En effet, si cette deuxième partie, c'est-à-dire notre score d'OG nouvellement créé, s'avère corrélée à la première partie , nous pourrons donc par la suite utiliser le score d'OG de façon autonome, sans avoir à utiliser le score de Girerd.

III.2.2.1.2. <u>Le score de communication médecin-malade (annexe 2) :</u>

Son élaboration a fait l'objet d'une étude préliminaire, menée par la même équipe de chercheurs (médecins, statisticiens, psychologues de la santé,

pharmaciens, internes en médecine, et étudiants en psychologie). Une revue de la littérature concernant les différents scores et échelles existants a été effectuée. À partir de ces échelles ont été extraits les items pertinents pour évaluer la CMM (annexe 3 : Méthodologie d'élaboration du score de CMM), ce qui a donné lieu à un nouveau questionnaire de CMM (annexe 2).

III.2.2.1.3. <u>Le questionnaire général : données sociodémographiques et score de satisfaction (annexe 4)</u>

Il a été élaboré pour permettre d'éliminer certains biais dus aux données sociodémographiques et de réaliser des analyses en sous-groupes.
De plus l'analyse de la satisfaction des patients (de façon globale et sur certains détails de la prise en charge) lors de leur passage aux urgences est indispensable pour permettre la validation des modifications de pratiques professionnelles.
Il sera ainsi recherché des liens de corrélation entre satisfaction et CMM ou OG.

III.2.2.2. Recrutement des investigateurs

Prise de contact avec le cadre administratif et le médecin chef du service des Urgences de la Clinique Mutualiste de Grenoble pour leur présenter le projet.
Puis courte formation et remise d'une lettre explicative sur l'étude aux médecins urgentistes susceptibles de participer à l'inclusion des patients (annexe 5).

III.2.2.3. Inclusion des patients

Pendant la période d'inclusion, lorsqu'un patient consultera pour une des 2 situations suivantes : entorse de cheville ou pyélonéphrite aiguë, le médecin investigateur lui proposera de participer à l'étude et lui remettra le formulaire d'information et de non-opposition (annexe 6), expliquant le déroulement et l'intérêt de celle-ci. S'il est d'accord, les médecins rempliront, pour chaque patient, une fiche d'inclusion (annexe 7) qui sera récupérée par les coordonnateurs de l'étude sur place, dans un délai maximum de 3 jours. Cette fiche contiendra tous les éléments d'identification nécessaires à un suivi de qualité. Ces données nominatives ne seront pas

informatisées ; elles serviront uniquement au suivi téléphonique et seuls les résultats seront intégrés à la base de données de façon anonyme.
En cas de refus de participer à l'étude, la case refus de participation sera cochée sur la fiche d'inclusion qui sera remplie le jour de la consultation et récupérée également par les coordonnateurs dans un délai maximum de 3 jours suivant la consultation.

III.2.2.4. Enquête téléphonique

Entre 7 et 10 jours après la consultation du patient aux urgences, nous l'interrogerons par téléphone à l'aide du questionnaire général (annexe 4) et des questionnaires des scores d'OG (annexe 1) et de CMM (annexe 2).
S'il n'est pas joignable la première fois, nous tenterons de le joindre à nouveau 2 fois (nous lui aurons demandé au préalable, lors de l'inclusion, sur quelle plage horaire il désire être contacté). En dernier recours nous appellerons un de ses proches (si mentionné sur la fiche d'inclusion). En cas d'échec le patient sera considéré comme perdu de vue.

III.2.3. Procédures d'investigation menées et différences par rapport à la prise en charge habituelle

Aucune différence par rapport à la prise en charge habituelle. Seul un contact téléphonique avec le patient est prévu. Une lettre d'information lui demandant son autorisation lui sera remise (annexe 6).

III.2.4. Tableau synoptique

Phase préparatoire de 6 mois	Élaboration des questionnaires<br>Prise de contact et formation brève des médecins investigateurs
J0	Consultation aux urgences et inclusion ou non du patient
J0 à J7	Retour du patient à domicile
J7 à J10	Appel du patient et enquête téléphonique avec questionnaire élaboré, recueil de données
M2-M4	Analyses statistiques des résultats et interprétation

IV. <u>POPULATION ÉTUDIÉE</u>

IV.1. Modalités de recrutement des sujets

Patient majeur qui consulte au service des Urgences de la Clinique Mutualiste de Grenoble pour l'une des 2 pathologies suivantes : l'entorse de cheville ou la pyélonéphrite aiguë.

IV.2. Critères d'inclusion

Seront proposés pour l'étude les sujets répondant à chacun des critères suivants :

- Patient majeur
- Patient pouvant être joint par téléphone dans les 7 à 10 jours qui suivent.

IV.3. Critères d'exclusion

Ne pourront pas être inclus les sujets répondant à au moins un des critères suivants :

- Patient analphabète
- Patient refusant le suivi téléphonique
- Patient porteur d'une déficience visuelle ou auditive

- Patient non francophone
- Personne privée de liberté par décision judiciaire ou administrative, personne faisant l'objet d'une mesure de protection légale.
- Patient dont la suite de la prise en charge conduit à l'hospitalisation

V. <u>CHOIX DES PATHOLOGIES</u>

Le nombre de 2 pathologies paraît être un bon compromis entre l'exhaustivité (évaluation de 2 situations cliniques aiguës différentes : l'une concernant de la traumatologie et l'autre la médecine infectieuse) et la faisabilité de l'étude.
Nous avons choisi 2 pathologies fréquemment rencontrées dans des services d'urgences et pour lesquelles la prise en charge et les conseils donnés sont doubles : médicamenteux et non médicamenteux (règles hygiéno-diététiques et proposition d'un suivi et d'examens complémentaires), afin que les deux versants de l'observance thérapeutique puissent être évalués.

VI. <u>VARIABLES MESUREES ET METHODES DE MESURE</u>

Réponses aux questionnaires lors de l'enquête téléphonique entre J7 et J10 suivant l'inclusion et calcul des scores.

- <u>Score d'observance</u> (annexe 1) : Le score d'observance médicamenteuse selon Girerd est coté de 0 à 5. Le score d'observance globale proposé par notre équipe comprend 4 sous-dimensions cotées chacune de 1 à 4 selon une échelle de Likert. Le score étant est la médiane des sous-dimensions.

- <u>Score de communication médecin-malade</u> coté sur 60 points (annexe 2),

- Mesure des propriétés intrinsèques (coefficient de Cronbach) et extrinsèques des scores d'observance et de CMM

- <u>Score de satisfaction</u> (annexe 4) : calculé à partir des questions 6 à 10 du questionnaire général, afin d'obtenir un score total coté sur 20 points (échelle de Likert de 1 à 4).

- Test de corrélation entre le score de communication médecin-malade, le score d'observance global et le score de satisfaction.

VII. RECUEIL ET GESTION DES DONNÉES

VII.1. Les données nominatives

Les informations concernant les patients inclus seront recueillies par l'investigateur et notées sur la fiche d'inclusion (annexe 7) : nom, prénom et coordonnées téléphoniques.

Pour chaque patient, un code d'identification est construit : il correspond aux 2 premières lettres de son nom suivies de la première lettre de son prénom.

Les coordonnateurs récupèreront l'ensemble des fiches à la Clinique Mutualiste, dans un délai maximum de 3 jours suivant la consultation.

Ceux-ci serviront à contacter les patients et seront archivés dans un dossier spécifique au protocole, qui constituera les données sources.

Les données nominatives ne donneront lieu à aucun traitement informatisé.

Seuls les codes d'identification des patients seront utilisés pour le traitement informatique des données de l'enquête téléphonique.

VII.2. Les questionnaires

Les données sont collectées au moyen d'une enquête téléphonique, menée par les coordonnateurs : Amélie DUVERT et Laure GONNET, internes de médecine générale en fin de cycle. Le support utilisé correspond à des questionnaires (annexes 1, 2, et 4), ils seront remplis au cours de l'appel téléphonique.

VIII. ANALYSE STATISTIQUE DES PARAMÈTRES MESURÉS

VIII.1. Calcul du nombre de sujets

L'objectif est l'obtention d'un minimum de 75 questionnaires remplis par pathologie. Étant donné que nous testons 2 fiches, il faudra 150 questionnaires.

Pour obtenir 150 questionnaires, avec un nombre de perdus de vue estimé à 25%, il faudra inclure 200 patients soit 100 par pathologie.

VIII.2. Responsables des analyses

- Rédacteur du plan d'analyse statistique : Professeur J-L Bosson
- Personnes chargées de réaliser l'analyse : les coordonnateurs, Amélie DUVERT et Laure GONNET

VIII.3. Lieu d'analyse des données et logiciels utilisés

Centre d'Investigation Clinique du CHU de Grenoble.

Les données seront saisies via une interface de saisie ad hoc. Un contrôle qualité sur 100 % des données et 10% des individus sera effectué.

L'analyse statistique et l'archivage de la base de données (après procédure de gel de base) se fera sous la responsabilité du Professeur Jean Luc Bosson. L'analyse statistique se fera avec le logiciel STATA Version 10 OSX (StataCorp LP 4905 Lakeway Drive College Statio, Texas 77845 USA).

VIII.4. Méthode d'analyse des données

L'analyse descriptive porte sur l'ensemble des variables recueillies. Elle utilisera les descripteurs visuels (nombre et fréquence pour les variables quantitatives, médiane et inter quantité pour les variables continues).Les tests statistiques de corrélation entre scores seront faits avec le risque d'erreur de première espèce usuel alpha = 0.05. Les critères principaux seront mesurés avec calcul de l'intervalle de confiance à 95%.

Pour chaque score une ACP et le calcul du coefficient de Cronbach permettront de caractériser ces scores.

IX. CALENDRIER PRÉVISIONNEL DE L'ÉTUDE

- Durée de la phase préparatoire : élaboration des questionnaires : printemps – été 2013
- Durée de l'étude par patient : 7 à 10 jours
- Durée totale de l'étude : 4 mois
- Date prévue pour le début des inclusions : octobre 2013
- Date prévue pour la fin des inclusions : décembre 2013
- Date prévue pour la fin de l'étude : janvier 2014

- Date prévue pour la phase secondaire de l'étude (Évaluation de l'impact des FIP sur la CMM et l'OG) : année 2014

X. ARRÊT PRÉMATURÉ DE L'ÉTUDE

X.1. Critères d'arrêt de l'étude pour un sujet qui y participe

- Sujet non joignable après 3 essais de contact téléphonique
- Sujet refusant de répondre au questionnaire
- Sujet décédé

X.2. Arrêt de l'étude par le coordonnateur

Lorsque le coordonnateur aura reçu un maximum de 100 fiches d'inclusion pour une pathologie donnée, les investigateurs seront avertis de l'arrêt de l'inclusion pour cette pathologie, afin d'assurer la représentativité des deux situations.

X.3. Arrêt de l'étude par l'investigateur

En cas d'impossibilité d'effectuer le protocole, l'investigateur peut arrêter l'étude en accord avec le coordonnateur.

XI. ASPECTS MATÉRIELS ET LÉGAUX

XI.1. Information du patient

Conformément aux Bonnes Pratiques Cliniques et aux dispositions légales en vigueur, tout sujet présélectionné sera préalablement informé par l'investigateur des objectifs de l'étude, de sa méthodologie, de sa durée, de ses contraintes. Il sera notamment précisé au patient qu'il est entièrement libre de refuser de participer à l'étude à tout moment sans encourir aucune responsabilité ni aucun préjudice de ce fait. Un document résumant les renseignements donnés par l'investigateur lui sera remis (annexe 6).

XI.2. Secret professionnel, confidentialité

L'investigateur sera tenu au respect du secret professionnel. Les données recueillies, y compris les résultats des analyses, seront rendues anonymes par tout moyen approprié. Les coordonnateurs seront soumis aux mêmes obligations de secret professionnel que les investigateurs.

Le présent document et ses annexes seront remis aux investigateurs à titre confidentiel et ne devront être remis ou communiqués qu'aux personnes nommément impliquées dans l'essai avec l'accord ou à la demande du coordonnateur.

XI.3. Financement de l'étude

Aucun financement.

Soutien méthodologique et statistique du CIC du CHU de Grenoble.

XI.4. Comité d'éthique

Cette étude est observationnelle, évaluation de pratiques sans procédure particulière de surveillance (le suivi téléphonique n'étant pas considéré comme interventionnel pour le patient). La soumission à une CPP et à l'autorité compétente n'est donc pas requise.

Elle a été soumise au CECIC (Comité d'Ethique des Centres d'Investigation Clinique de l'inter région Rhône Alpes Auvergne). Le CECIC examine et donne un avis consultatif concernant l'aspect éthique de la conduite d'un protocole à la condition que celui-ci ne relève pas de la loi française sur la recherche biomédicale.

XI.5. Amendements au protocole

Toute modification substantielle du protocole fera l'objet d'un amendement soumis pour avis au CECIC.

XI.6. Anonymat des sujets participant à l'étude

Les questionnaires remplis ne porteront que les initiales du patient (deux premières lettres du nom et la première lettre du prénom) et un numéro d'anonymat. Seul ce numéro sera informatisé.

XI.7. Publications

Toutes les données recueillies au cours de cette étude sont la propriété du promoteur de l'étude et ne peuvent être communiquées en aucun cas à une tierce personne sans l'accord écrit de l'investigateur.

Toute publication ou communication (orale ou écrite) sera décidée d'un commun accord entre les investigateurs et respectera les recommandations internationales: "Uniforms Requirements for Manuscripts Submitted to Biomedical Journals"

XI.8. Archivage

L'ensemble des dossiers de l'étude sera archivé pour une durée de 15 ans, sous la responsabilité des coordonnateurs.

Le protocole signé et les fiches d'inclusion devront être conservés par les investigateurs pendant la durée minimale de 15 ans à compter de la fin de l'étude.

Les coordonnateurs organiseront, le stockage dans des locaux appropriés les documents suivants :

- Protocole avec annexes, amendements.
- Fiches d'inclusion et questionnaires (originaux) avec documents annexes.
- Toutes les pièces administratives et correspondances liées à l'étude.
- Rapport d'étude.

XII. DATE ET SIGNATURES

Ce protocole a été lu et approuvé à la date notée en en-tête

Investigateurs coordonnateurs signataires

Dr Mélanie SUSTERSIC

Pr Jean-Luc BOSSON

Investigateurs coordonnateurs

Amélie DUVERT

Laure GONNET

XIII. REFERENCES BIBLIOGRAPHIQUES

1. Loi HSPT Article 59 - Loi n°2009-879 du 21 juillet 2009 portant sur la réforme de l'hôpital et relative aux patients, à la santé et aux territoires
2. Arrêté du 26 février 2013 fixant la liste des orientations nationales du développement professionnel continu des professionnels de santé pour l'année 2013
3. Haynes R.B, Taylor D.W et Sackett D.L. Compliance in Health Care. 1979. MD: Johns Hopkins University Press, Baltimore: 1-15
4. Baudrant M. Réflexions sur la place du pharmacien dans l'éducation thérapeutique du patient. Journal de pharmacie clinique. 2008;27(4):201‑4.
5. World Health Organization. (2003). Adherence to long-term therapies, evidence for action. Geneva.
6. Rapport de l'OMS-Europe de 1998 : Therapeutic patient education. Continuing education programmes for health care. Providers in the field of prevention of chronic diseases. World Health Organization. 1998, 77 p
7. Haute Autorité de Santé (HAS) et Institut National de Prévention et d'Education pour la Santé (INPES) : "Structuration d'un programme d'éducation thérapeutique du patient dans le champ des maladies chroniques", 2007.
8. Article 35 du code de Déontologie
9. Article R.4127-35 du Code de Santé Publique
10. Wu, E. Q., Guerin, A., Yu, A. P., Bollu, V. K., Guo, A., & Griffin, J. D. (2010). Retrospective real-world comparison of medical visits, costs, and adherence between nilotinib and dasatinib in chronic myeloid leukemia. Curr Med Res Opin, 26 (12), 2861–2869.
11. Morin M. De la recherche à l'intervention sur l'observance thérapeutique : contributions et perspectives des sciences sociales. L'observance aux traitements contre le VIH/Sida : Mesures, déterminants, évolution. Paris, ANRS
12. Ley P. Improving patients'understanding, recall, satisfaction and compliance. In: Broome A, ed.Health psychology: processes and applications, 2e ed.London: Chapman, 1995

13. Tarquinio C., Fischer, G.N. & Grégoire, A. La compliance chez des patients atteints par le VIH : Validation d'une échelle française et mesure de variables psychosociales. Revue Internationale, 2000.
14. Fuertes JN, Mislowack A, Bennett J, Paul L, Gilbert TC, Fontan G, et al. The physician-patient working alliance. Patient Educ Couns. avr 2007;66(1):29‑36.
15. Ciechanowski P, katon W, Russo J et al. The patient-provider relationship: attachment theory an adherence in diabetes. Am J Psychiatry 2001;158:29-35
16. Rainer S. Beck et al. Physician Patient Communication in the Primary Care Office: A Systematic Review. JABFP January–February 2002 Vol. 15 No. 1
17. Kennedy A, Nelson E, Reeves D, Richardson G, Roberts C, Robinson A, et al. A randomised controlled trial to assess the impact of a package comprising a patient-orientated, evidence-based self-help guidebook and patient-centred consultations on disease management and satisfaction in inflammatory bowel disease. Health Technol Assess. 2003;7(28):iii, 1‑113.
18. Sustersic M, Meneau A, Dremont R, Bosson J-L. Fiches d'information patient : quelle méthodologie ? La Revue du praticien. Médecine générale. 2007 Déc 4;790:1167-68.
19. Sustersic M, Meneau A, Bosson JL; Elaboration de fiches d'information pour les patients en médecine générale. supplément- Rev du Prat 2008; 58.
20. Southall AC, Harris VV. Patient ED turnaround times: a comparative review. Am J Emerg Med. mars 1999;17(2):151‑153.
21. Motifs et trajectoires de recours aux urgences hospitalières, rapport d'une enquête de la DREES en ligne sur www.sfmu.org/documents/ressources/referentiels/er215.pdf
22. Thèse de médecine générale : Parcours de soins et motifs de recours aux urgences hospitalières en ligne sur www.bichat-larib.com/publications.../3327_MEUNIER_Laure_these.pdf
23. Girerd X, Hanon O, Anagnostopoulos K, et al. Assessment of antihypertensive compliance using a self-administered questionnaire: development and use in a hypertension clinic.*Presse Med.* 2001;16-23; 30 (21): 1044-8.
24. Ruiz MA, Pardo A, Rejas J, Soto J, Villasante F, Aranguren JL. Development and validation of the « Treatment Satisfaction with Medicines Questionnaire » (SATMED-Q). Value Health. oct 2008;11(5):913‑926.

25. Care Coordination Measures Atlas [Internet]. 2011 [cité 10 sept 2013]. Disponible sur: http://www.ahrq.gov/professionals/systems/long-term-care/resources/coordination/atlas/index.html

ANNEXE 1

SCORE d'OBSERVANCE

Merci de répondre aux affirmations suivantes :

Votre médecin vous a-t-il prescrit des médicaments ? OUI ☐ NON ☐
Si non, passer directement aux questions II.

A- Questionnaire d'observance médicamenteuse de Girerd

1. Depuis la consultation, avez-vous oublié de prendre vos médicaments ?
   OUI ☐ NON ☐
2. Depuis la dernière consultation, avez-vous été en panne de médicament (vous n'êtes pas allez les chercher à la pharmacie ou n'en aviez pas assez) ?
   OUI ☐ NON ☐
3. Vous est-il arrivé de prendre votre traitement avec du retard par rapport à l'heure souhaitée ?
   OUI ☐ NON ☐
4. Vous est-il arrivé de ne pas prendre votre traitement parce que certains jours votre mémoire vous fait défaut ?
   OUI ☐ NON ☐
5. Vous est-il arrivé de ne pas prendre votre traitement parce que vous aviez l'impression que votre traitement vous fait plus de mal que de bien ?
   OUI ☐ NON ☐
6. Pensez-vous que vous avez eu trop de traitements à prendre ?
   OUI ☐ NON ☐

B- Questionnaire d'observance globale :

I- Observance des prescriptions médicamenteuses

Les questions qui suivent concernent les jours qui ont suivi la consultation :

1. Avez-vous pris l'ensemble du traitement proposé ?
   1 (non) 2 (plutôt non) 3 (plutôt oui) 4 (oui, tout à fait)

   Si non ou plutôt non :

   Il vous est arrivé de ne pas prendre l'un ou plusieurs de vos médicaments :

- Parce que vous avez oublié : oui non
- Parce que le traitement est trop complexe: oui non
- A cause d'effets secondaires: oui non
- Parce vos médicaments vous font plus de mal que de bien : oui non
- Parce vous pensiez qu'il n'était pas utile ou adapté: oui non
- Parce que vous sentiez déjà une amélioration : oui non
- Parce que vous n'êtes pas allé chercher les médicaments à la pharmacie : oui non

Si oui : pourquoi : ……………………………………………………………………………

2. Avez-vous respecté les doses prescrites ?

1 (non) 2 (plutôt non) 3 (plutôt oui) 4 (oui, tout à fait)

3. Avez-vous respecté les modalités de prises (l'heure, à jeun, avant les repas) ?

1 (non) 2 (plutôt non) 3 (plutôt oui) 4 (oui, tout à fait)

4. Avez-vous pris des médicaments autres que ceux prescrits pas votre médecin ?

1 (non) 2 (plutôt non) 3 (plutôt oui) 4 (oui, tout à fait)

x-1- Question subsidiaire de concordance :

D'une manière générale, est-ce une décision volontaire de votre part d'avoir pris ou non votre traitement :

1 (non) 2 (plutôt non) 3 (plutôt oui) 4 (oui, tout à fait)

II- Observance des prescriptions non médicamenteuses (examens complémentaires)

Votre médecin vous a-t-il prescrit des examens complémentaires et/ou proposé un suivi et/ou orienté chez un spécialiste ? OUI ☐ NON ☐

Si non, question non comptabilisée.

Si oui : les avez-vous réalisés ?

(Pas du tout) 1 2 3 4 (Complètement)

III- Observance des consignes/ conseils hygiéno-diététiques et de conduite à tenir selon l'évolution

1. Votre médecin vous a-t-il donné des conseils à respecter ? OUI ☐ NON ☐

(cf.liste ci-dessous pour vous aider)

Par exemple, conseils concernant :

- L’alimentation et/ ou la gestion du poids
- Vos activités habituelles
- L’exercice physique
- Le tabagisme et l’arrêt du tabac
- La consommation d’alcool (ex : diminution si consommation excessive> 2 verres/ jour et % de réussite de l’intention)
- La consommation d’eau (adaptation de la consommation d’eau aux besoins et % de réussite de l’intention).
- Les moyens d’éviter l’aggravation des symptômes, la récidive, la transmission
- Quand reconsulter

Si non : question non comptabilisée.

Si oui :

1- Avez-vous appliqué les conseils donnés et/ou modifié certaines habitudes suite à la consultation*?*

(Pas du tout) 1 2 3 4 (Complètement)

2- Certaines informations données par votre médecin pouvaient-elles intéresser vos proches ?

OUI ☐ NON ☐

Si non, question non comptabilisé.

Si oui, leur avez-vous transmis ces informations ?

(Pas du tout) 1 2 3 4 (Complètement)

x-2- ***Question subsidiaire de concordance :***

D’une manière générale, est-ce une décision volontaire de votre part d’avoir suivi ou non les conseils donnés par votre médecin ?

1 (non) 2 (plutôt non) 3 (plutôt oui) 4 (oui, tout à fait)

IV- Comportement de consommation de soins

Après la consultation, avez-vous eu besoin d’un nouvel avis médical pour le même problème ?

1 (non) 2 (plutôt non) 3 (plutôt oui) 4 (oui, tout à fait)

Si oui :

- Avez-vous consulté à nouveau votre médecin ? OUI ☐ NON ☐
- Avez-vous consulté un autre médecin ? OUI ☐ NON ☐
- Avez-vous consulté un service d’urgences ? OUI ☐ NON ☐
- Avez-vous appelé le centre 15 concernant le même problème ? OUI ☐ NON ☐

Selon vous, à quel point le fait d'avoir ou non reconsulté pour ce même motif de consultation était en adéquation avec l'attitude recommandée par votre médecin?

(Pas du tout) 1 2 3 4 (complètement)

**** x-3-Question subsidiaire de concordance :***

D'une manière générale, est-ce une décision volontaire de votre part d'avoir suivi ou non les recommandations de votre médecin sur « quand reconsulter » ?

1 (non) 2 (plutôt non) 3 (plutôt oui) 4 (oui, tout à fait)

SYSTEME de COTATION :

Le score d'observance globale proposé par notre équipe comprend 4 sous-dimensions cotées chacune de 1 à 4 selon une échelle de Likert. Le score étant est la médiane de sous-dimensions. Total de 4 à 16/ 16

- L'observance médicamenteuse ;
- L'observance des prescriptions non médicamenteuses ;
- L'observance des conseils hygiéno-diététiques ;
- L'observance de recours au système de soins ;

ANNEXE 2

Questionnaire évaluant la communication médecin malade

1-Le médecin vous a-t-il écouté attentivement pendant la consultation ?

□Non □ Plutôt non □ Plutôt oui □ Oui

2-Le médecin vous a-t-il interrompu pendant que vous parliez ?

□ Non □ Plutôt non □ Plutôt oui □ Oui

3- Le médecin vous a-t-il encouragé à vous exprimer ?

□Non □ Plutôt non □ Plutôt oui □ Oui

4- Le médecin vous a-t-il bien examiné ?

□ Non □ Plutôt non □ Plutôt oui □ Oui

5- Vous-êtes-vous senti compris par le médecin ?

□ Non □ Plutôt non □ Plutôt oui □ Oui

6- Est-ce que le médecin s'est exprimé de façon compréhensible ?
□ Non □ Plutôt non □ Plutôt oui □ Oui

7- Avez-vous eu l'impression d'avoir eu toutes les informations nécessaires ?
□ Non □ Plutôt non □ Plutôt oui □ Oui

8- Le médecin vous a-t-il expliqué les avantages et inconvénients du traitement ?

□ Non □ Plutôt non □ Plutôt oui □ Oui

9- Le médecin vous a-t-il impliqué dans la prise de décision ?

□ Non □ Plutôt non □ Plutôt oui □ Oui

10- D'après vous le médecin a-t-il eu une attitude et un discours rassurants ?

□ Non □ Plutôt non □ Plutôt oui □ Oui

11- D'après vous, le médecin a-t-il été globalement respectueux ?
□ Non □ Plutôt non □ Plutôt oui □ Oui

- Respect de votre intimité ? □ oui □ non
- Respect de vos croyances ? □ oui □ non
- Garde confidentielles les informations données ? □ oui □ non
- Ne porte pas de jugement ? □ oui □ non
- Respect de votre corps lors de l'examen clinique □ oui □ non

12- Avez-vous confiance en ce médecin ?
□ Non □ Plutôt non □ Plutôt oui □ Oui

13- D'après vous, le médecin vous a-t 'il dit toute la vérité ?
□ Non □ Plutôt non □ Plutôt oui □ Oui

14- Le médecin s'est-il assuré que vous aviez bien compris ses explications?

□ Non □ Plutôt non □ Plutôt oui □ Oui

15- Le médecin a-t 'il répondu à toutes vos attentes et/ou préoccupations?
□ Non □ Plutôt non □ Plutôt oui □ Oui

SCORE TOTAL : / 60

Chacun des items est coté selon la réponse 1, 2, 3 ou 4.
Score côté sur 60 avec un score minimum à 15/60 et un maximum à 60/60

ANNEXE 3

Méthodologie d'élaboration du questionnaire de communication médecin-malade

Objectif : Élaboration d'une échelle et de son score évaluant la qualité de la communication médecin malade à partir d'une revue de la littérature

Travail universitaire effectué avec l'appui méthodologique du CIC (Pr JLBosson), du CNRS (équipe ThEMAS) et de la faculté de psychologie (Dr Aurélie Gauchet, psychologie de la Santé).

Méthodologie :

La première étape a consisté en une recherche bibliographique sur les bases de données « pubmed » et « psychinfo » ainsi que les sites institutionnels français (HAS), anglais (NHS) et américain (AHRQ).
Les mots clés étaient « physician-patient relations », « psychometrics », « questionnaires », « scale », « communication ».

Pour élaborer une échelle multidimensionnelle évaluant la qualité de la communication médecin malade, nous nous sommes appuyés sur le modèle de communication suivant: dans un contexte donné commun, émission d'une information par le médecin avec des mots et un discours intelligible -> réception par le patient qui confirme au médecin que le message a bien été reçu. Ce qui peut être résumé de la manière suivante : émission -> information -> réception-> feedback.

Les échelles ont été sélectionnées selon les critères d'inclusion suivants : échelles validées ; en langue Française ou Anglaise ; évaluant la communication médecin malade (échelles de communication médecin malade, de relation médecin malade, d'écoute, de confiance, d'empathie).
Dans notre choix de modèle théorique, les conséquences de la communication sur le comportement (échelles d'observance), le psychisme (échelles de satisfaction, anxiété, dépression, auto efficacité personnelle etc.) ou les symptômes du patient (échelles de douleur…etc.) ont été considérées comme étant extrinsèques à la communication médecin malade. Les échelles mesurant ces dimensions ont donc été exclues. Les autres critères d'exclusion étaient : les échelles spécifiques des pathologies chroniques ; celles mesurant des dimensions hors champ de la consultation (organisation du système de soins, temps d'attente dans la salle d'attente…etc.). Au total, vingt échelles ont été retenues.

La seconde étape a consisté à extraire à partir des échelles retenues tous les items pertinents sur le sujet sans préjuger des dimensions auxquelles ils appartenaient. Ensuite, lorsque deux ou plusieurs items étaient redondants, un seul a été retenu, ce qui nous a permis d'aboutir à un questionnaire à la fois exhaustif et synthétique. Cette démarche (inverse par rapport à la démarche communément utilisée qui aurait été de choisir un modèle théorique en psychologie, de définir ensuite les dimensions

caractérisant le modèle et enfin de rédiger les items caractérisant chacune des dimensions) a permis d'éviter plusieurs écueils.
Tout d'abord celui de créer une énième échelle parmi tant d'autres sans prendre en compte les travaux antérieurs réalisés sur le sujet. Ensuite celui d'avoir des items chevauchant potentiellement plusieurs dimensions.
En effet, les dimensions ayant des définitions variables selon les équipes de chercheurs, le choix d'un modèle théorique implique une part d'arbitraire. Aucun modèle théorique n'étant parfait, il est forcément réducteur de choisir un modèle plutôt qu'un autre.
Notre classification par dimensions a eu pour seul objectif de faciliter le tri des items et aucunement d'attribuer à chaque item une dimension. Notre échelle et/ou questionnaire fonctionne donc indépendamment des dimensions qui le composent.

La troisième étape a consisté à reformuler les items afin que les réponses aux questions puissent être mesurées sur une échelle de Likert allant de 1 à 4.

Une étude qualitative réalisée sur une vingtaine de patients est actuellement en cours.
Elle consiste à demander au patient pour chacun des items :
- une reformulation de l'item pour s'assurer qu'il l'a bien compris;
- savoir si l'item lui paraît pertinent par une question ouverte;
- savoir si l'item englobe bien tous les aspects qu'il était censé englobé lorsqu'il a été sélectionné, afin de ne pas omettre de notion.

Elle a pour but de vérifier la bonne compréhension du questionnaire par les patients avant d'aborder la seconde étape, quantitative, qui se fera sur environ 200 patients, afin de valider l'échelle et ses propriétés psychométriques.

Références bibliographiques

- Atlas AHRQ : http://www.childhealthdata.org/docs/drc/ahrq-care-coordination-atlas-dec-2010.pdf

- Mack JW et al. Measuring therapeutic alliance between oncologists and patients with advanced cancer: the Human Connexion Scale. Cancer. 2009 Jul

- Jane Ogden. Psychologie de la santé. Edition De Boeck 2004

Claude Richard et Marie-Thèrèse Lussier. La communication professionnelle en santé. Édition Erpi. 2002

- Rainer S. Beck et al. Physician Patient Communication in the Primary Care Office: A Systematic Review. JABFP January–February 2002 Vol. 15 No. 1

ANNEXE 4

Questionnaire général

Code d'identification du patient :

Date de consultation aux urgences :

Date(s) d'appel téléphonique :

Nous souhaitons recueillir votre opinion sur votre dernière consultation aux urgences. Cet entretien va durer environ 15 minutes.
Nous vous remercions du temps que vous allez prendre pour répondre à cette enquête.
Vos réponses sont anonymes.

A/ Données sociodémographiques

Tout d'abord, quelques questions pour mieux vous connaître :

1- Quel âge avez-vous ?

2- Quel est votre niveau d'études ?
□ Collège □ Bac □ Bac +2 □ Études supérieures après Bac +2 □ autres

3- Travaillez-vous dans le milieu médical ?
□ Oui □ Non

4- A quelle catégorie socio-professionnelle appartenez-vous ?

5- Quelle est votre situation familiale : célibataire / en couple / marié ;
et nombre d'enfants :

B/ Score de Satisfaction :

Suite à votre consultation dans le service d'urgences :

6- Diriez-vous que vous êtes satisfait des conditions d’accueil, hors paramètres médicaux (accueil, nourriture, délais d’attente) dont vous avez bénéficié ?
□ Non □ Plutôt non □ Plutôt oui □ Oui

7- Diriez-vous que vous êtes satisfait des soins reçus et de la prise en charge par l’équipe paramédicale (infirmier(e)s, aides-soignants, ASH) ?
□ Non □ Plutôt non □ Plutôt oui □ Oui

8- Diriez –vous que vous êtes satisfait de la prise en charge par le médecin que vous avez rencontré ?
□ Non □ Plutôt non □ Plutôt oui □ Oui

9- De façon globale, diriez-vous que vous êtes satisfait de votre consultation aux urgences ?
□ Non □ Plutôt non □ Plutôt oui □ Oui

- Quels sont les éléments dont vous êtes satisfaits ?
………………………………………………………………………………
………………………………………………………………………………
……………………….
- Quels sont les éléments qui vous ont déplu ?
………………………………………………………………………………
………………………………………………………………………………
……………………….

10- Recommanderiez-vous ce service à votre famille et vos amis ?
□ Non □ Plutôt non □ Plutôt oui □ Oui

Chacun des items est coté selon la réponse : 1(non), 2, 3 ou 4 (oui).

Score coté sur 20 avec un minimum de 5/20 et un maximum de 20/20.

ANNEXE 5

Cher confrère,

Nous vous remercions de participer à cette étude, qui a plusieurs objectifs :
1/ Première étape (Hiver 2013) :

- Faire un **état des lieux des niveaux de satisfaction des patients, de communication médecin-malade, et d'observance thérapeutique** dans un service d'urgences;
- Valider deux scores (communication médecin-malade et observance thérapeutique) génériques, dans le but ensuite de pouvoir les utiliser comme outil d'évaluation de la qualité de toute consultation.

2/ Deuxième étape (Hiver 2014) visant à améliorer la prise en charge des patients, à l'aide d'un outil : les **Fiches Information Patients** (FIP).

- Évaluer la faisabilité de l'usage de ces FIP dans un service d'urgence ;
- Évaluer leur impact sur la communication médecin-malade et l'observance thérapeutique.

Cette étude s'inscrit ainsi dans une démarche **d'Evaluation des Pratiques Professionnelles et de Développement Professionnel Continu**, qui est rendu obligatoire par la loi HPST du 21 Juillet 2009.

Durant cette période, nous vous remercions de bien vouloir nous aider en suivant la démarche ci-dessous :

Patient répondant aux critères d'inclusion :
Patient majeur capable, lettré, joignable par téléphone dans les dix jours qui suivent,
Consultant pour : Pyélonéphrite Aiguë ou Entorse de Cheville

↓

Consultation Habituelle
Remise de la lettre d'information au patient et explication de l'étude

↙ ↘

**Accord** du patient pour être contacté par téléphone	**Refus** du patient de participer à l'étude
↓	↓
Remplir la fiche d'inclusion<br>**Avec les données** nominatives du patient<br>En cochant la fiche distribuée	Remplir la fiche d'inclusion<br>**Sans les données** nominatives du patient<br>En cochant « refus de contact téléphonique »

↘ ↙

Mettre la fiche d'inclusion dans la corbeille prévue à cet effet

Vous recevrez bien sûr les résultats de cette étude.
Si vous avez la moindre interrogation, vous pouvez nous joindre au 06 64 36 42 77 (Laure), ou au 06 72 31 23 94 (Amélie).
Merci encore pour votre participation à cette étude.

ANNEXE 6

FORMULAIRE D'INFORMATION ET DE NON-OPPOSITION

Document constitué en application du Code de Santé Publique.

Madame, Monsieur,

Nous vous proposons de participer à l'étude « Etat des lieux dans un service d'Urgences dans un but d'Amélioration des Pratiques Professionnelles ».

Le médecin que vous avez rencontré ce jour vous a proposé de participer à une étude concernant votre consultation aux urgences.

But de l'étude :

Nous réalisons une étude pour évaluer la qualité des consultations aux urgences (en termes de communication médecin-malade, et de satisfaction des patients notamment).

Le but à terme serait de pouvoir proposer un outil visant à améliorer ces paramètres (via l'utilisation de Fiches d'Information destinés aux patients).

Nous vous proposons de participer à cette étude. En effet, votre avis est essentiel pour évaluer la qualité de votre prise en charge, et pouvoir l'améliorer.

Déroulement de l'étude :

Votre avez consulté aujourd'hui aux urgences pour une pathologie donnée, le médecin que vous avez rencontré vous a prescrit un traitement et/ou des conseils à suivre.

Vous serez ensuite contacté(e) par téléphone dans une dizaine de jours par un des médecins coordonnateurs de l'étude. Cela durera une dizaine de minutes. Cet appel téléphonique garantit le secret médical. En cas de répondant intermédiaire, aucune information médicale ne sera donnée.

Votre participation ne modifie en rien les pratiques médicales vous concernant (examens, traitements).

Afin de pouvoir vous joindre, nous avons besoin de vos coordonnées. Nous vous demandons également les coordonnées d'un proche pour permettre aux coordonnateurs d'effectuer l'enquête si vous-même n'êtes pas en mesure de répondre.

Confidentialités des données vous concernant :

Dans le cadre de cette étude les règles usuelles de confidentialité seront respectées.

Les données médicales, vous concernant, seront centralisées dans un fichier informatique à des fins de traitement statistique. Elles resteront strictement confidentielles. Ces données

seront transmises au Centre d'Investigation Clinique du CHU de Grenoble ou aux personnes agissant pour son compte.
Suite à l'enquête téléphonique, vos coordonnées personnelles seront détruites. Vos réponses resteront anonymes. Elles se verront attribuer un code comportant un numéro et les initiales de votre nom et prénom, qui lui seul sera informatisé.

Conformément aux dispositions de la loi relative à l'informatique aux fichiers et aux libertés, vous disposez d'un droit d'accès et de rectification. Vous disposez également d'un droit d'opposition à la transmission de vos données couvertes par le secret professionnel susceptibles d'être utilisées et traitées dans le cadre de cette recherche, en contactant les médecins coordonnateurs de l'étude nommés ci-dessous.

Vous avez bien sûr le droit de refuser de participer à cette recherche, il vous suffit de remplir le bas de ce document et de le remettre au médecin qui vous prend en charge, ce refus ne changera en rien vos relations avec les médecins. Vous avez en outre la possibilité de vous y opposer à tout moment pour la raison de votre choix, sans encourir aucune responsabilité, sans atteinte à la qualité des soins que vous recevrez ultérieurement.

En coopérant à cette enquête, vous participerez à la recherche médicale. Vous aidez les médecins français à utiliser de nouveaux moyens d'information et nous vous en remercions.

Je soussigné(e) Mme, Mr…………………………

Ne souhaite pas participer à l'étude « Etat des lieux dans un service d'Urgence dans un but d'Amélioration des Pratiques Professionnelles ».
Aucune donnée concernant mon dossier médical ne sera prise en compte pour l'étude. J'ai bien compris que le fait de ne pas participer à l'étude ne changera en rien la façon dont je serai prise en charge.

Fait le ………………………………, à ………………………………,

Signature :

Nous vous remercions pour votre participation à cette étude

DUVERT Amélie et GONNET Laure
Internes en Médecine Générale
Coordonnateurs de l'étude

ANNEXE 7

FICHE d'INCLUSION

(à remettre dans la corbeille prévue à cet effet)

Date de la consultation d'inclusion :

Refus de contact téléphonique : ☐

(si case cochée, ne pas remplir les données nominatives)

Données nominatives du patient (non informatisées)

Nom :

Prénom :

Sexe :

Téléphone fixe patient: /__/__/__/__/__/__/__/__/__/__/

Téléphone portable patient : /__/__/__/__/__/__/__/__/__/__/

Téléphone d'un proche : /__/__/__/__/__/__/__/__/__/__/ (précisez : voisin, parent…)

Jour de la semaine et créneaux horaires préférés par le patient pour être contacté :

Code d'identification du patient :

2 premières lettres du nom du patient : /__/__/

Première lettre du prénom du patient : /__/

Pathologie concernée (entourer la mention utile)

- Entorse cheville
- Pyélonéphrite aigue
